# In vitro basal T-cell proliferation and HTLV-1 proviral load among HTLV-1 subjects co-infected with Hepatitis C and/or HIV-1

**DOI:** 10.1186/1742-4690-12-S1-P17

**Published:** 2015-08-28

**Authors:** Tatiane Assone, Tatiana Mitiko, Samara PC Gomes, Arthur Paiva, Michel Haziot, Jerusa Smid, Augusto Penalva de Oliveira, Philip J Norris, Jorge Casseb

**Affiliations:** 1 Laboratory of Dermatology and Immunedeficiencies, Department of Dermatology, University of São Paulo Medical School, Brazil; 2 Institute of Tropical Medicine of São Paulo, São Paulo, SP, Brazil; 3 Institute of Infectious Diseases “Emilio Ribas” (IIER) of São Paulo, São Paulo, SP, Brazil; 4 Blood Systems Research Institute, San Francisco, California, USA

## Background

HTLV-1 infection may is present among HCV or HIV-infected subjects and is associated with higher risk for HAM/TSP and other inflammatory diseases. Lymphocytes from about half of HTLV-1-infected subjects spontaneously proliferate in vitro, and how this phenomenon relates to symptomatic disease outcome and viral burden is poorly understood. Objective: Evaluate T-cell proliferation in vitro and HTLV-1 proviral load (PVL) among co-infected subjects.

## Material and methods

From 522 HTLV-1-infected individuals, who presented co-infection with HCV or HIV were selected during their outpatient visits to the HTLV clinic at the IIER in Sao Paulo. All the volunteers were invited to participate after reading and signing a consent form. Clinical data were obtained from medical records and interviews. PBMC from patients and controls, 2 x106 cells/ml in RPMI with 10% fetal calf serum were incubated at 37 C and 5% CO2 for 3 days in triplicate. Cells were pulsed with tritiated thymidine 18 h before harvest in a semi-automatic cell harvester and counted in a beta-counter. To determine the proviral load, the HTLV-1 DNA copy number was done by RT-PCR to the amount of the cellular albumin of the clinical sample, which was quantified in parallel. Results were expressed as HTLV-1 DNA copies/104 PBMCs. Statistical analysis was conducted using Kruskal-Wallis test.

## Results

From a total infected with HTLV-1, 123 (24%) subjects presented co-infection, where 81 had HIV-1 (15%), 40 had HCV (8%) and 37 had HIV-1 and HCV (7%). The basal T-cell proliferation show an increase among HCV/HTLV-1 subjects compared to patients with HTLV-1/HIV-1 co-infection and HTLV-1/ HIV-1/HCV (p=0.1). The HTLV-1 PVL was also highest in the HTLV-1/HCV subjects (p=0.6).

## Conclusion

We observed a significant increase of basal T-cell proliferation among HTLV-1/HCV co-infected. However, the presence of co-infection with HIV-1 may induce a down regulation at T-cell proliferation capacity. Support: FAPESP 2012/23397-0.

